# Improving therapeutic HPV peptide-based vaccine potency by enhancing CD4+ T help and dendritic cell activation

**DOI:** 10.1186/1423-0127-17-88

**Published:** 2010-11-22

**Authors:** Chao-Yi Wu, Archana Monie, Xiaowu Pang, Chien-Fu Hung, T-C Wu

**Affiliations:** 1 Department of Pathology, Johns Hopkins Medical Institutions, Baltimore, Maryland, USA; 2 Department of Obstetrics and Gynecology, Johns Hopkins Medical Institutions, Baltimore, Maryland, USA; 3 Department of Molecular Microbiology and Immunology, Johns Hopkins Medical Institutions, Baltimore, Maryland, USA; 4 Department of Oncology, Johns Hopkins Medical Institutions, Baltimore, Maryland, USA; 5 Department of Oral Diagnostic Service, Howard University, Washington DC, USA

## Abstract

**Background:**

Effective vaccination against human papillomavirus (HPV) represents an opportunity to control cervical cancer. Peptide-based vaccines targeting HPV E6 and/or E7 antigens while safe, will most likely require additional strategies to enhance the vaccine potency.

**Methods:**

We tested the HPV-16 E7 peptide-based vaccine in combination with a strategy to enhance CD4+ T help using a Pan HLA-DR epitope (PADRE) peptide and a strategy to enhance dendritic cell activation using the toll-like receptor 3 ligand, poly(I:C).

**Results:**

We observed that mice vaccinated with E7 peptide-based vaccine in combination with PADRE peptide and poly(I:C) generated better E7-specific CD8^+ ^T cell immune responses as well as significantly improved therapeutic anti-tumor effects against TC-1 tumors compared to E7 peptide-based vaccine with either PADRE peptide or poly(I:C) alone. Furthermore, we found that intratumoral vaccination with the E7 peptide in conjunction with PADRE peptide and poly(I:C) generates a significantly higher frequency of E7-specific CD8^+ ^T cells as well as better survival compared to subcutaneous vaccination with the same regimen in treated mice.

**Conclusions:**

The combination of PADRE peptide and poly(I:C) with antigenic peptide is capable of generating potent antigen-specific CD8+ T cell immune responses and antitumor effects in vaccinated mice. Our study has significant clinical implications for peptide-based vaccination.

## Introduction

Cervical cancer is the 2^nd ^leading cause of cancer deaths in women worldwide. The primary etiological factor in the development of cervical cancer is infection by human papillomavirus (HPV) [1]. HPV is one of the most common sexually transmitted diseases in the world. It is now known that cervical cancer is a consequence of persistent infection with high-risk type HPV [1-5]. HPV infection is a necessary factor for the development and maintenance of cervical cancer and thus, effective vaccination against HPV represents an opportunity to control cervical cancer (for reviews see [6,7].

Peptide-based vaccination has emerged as a potentially important strategy for the development of therapeutic HPV vaccination as they are considered to be safe, easy to produce, and stable [8,9]. The most important factor in the designing of therapeutic vaccines is the choice of target antigen. In the case of HPV, the early viral proteins such as E6 and E7 represent ideal target antigens since they are consistently expressed in a majority of cervical cancers and its precursor lesions and are essential for transformation [10]. The high-affinity H-2D^b^-restricted E7-specific CTL epitope aa49-57 (RAHYNIVTF) has been previously used in vaccination studies against HPV 16-transformed tumor cells [11]. These studies have shown that vaccination with the E7 peptide-based vaccine with incomplete Freud's adjuvant induced E7-specific CD8+ T cell immune responses which resulted in antitumor effects in a preclinical model [11]. This study suggests that with an appropriate strategy, such as selecting an appropriate adjuvant, it is feasible to enhance peptide-based vaccine potency. Thus, it is important to continue to identify strategies to enhance peptide-based vaccine potency that may potentially be suitable for clinical translation.

One strategy to enhance peptide-based vaccine potency is to induce CD4+ T helper cell immune responses. CD4^+ ^T helper cells are known to play an important role in the generation of CD8^+ ^T cell immune responses as well as memory T cell responses (for review see [12]). Thus, it is desirable to design an immunization regimen that is capable of generating antigen-specific CD4^+ ^T cells. Previously, a Pan HLA-DR epitope peptide (PADRE) has been described that is capable of binding to different MHC class II molecules with high-affinity [13]. PADRE peptides have been used in conjunction with other forms of vaccines to enhance vaccine potency in preclinical models [13-15]. PADRE peptides have also been used in clinical trials with minimal toxicity [16,17].

Another strategy to enhance the peptide-based vaccine potency is to activate dendritic cells via toll-like receptors (TLR). DC activation is a prerequisite to T cell priming and the generation of antigen-specific immune responses. In the presence of "alert" signals such as TLR ligands or inflammatory cytokines, DCs are stimulated to mature and differentiate into potent activators of antigen-specific T cells (for review, see [18]). Toll-like receptor 3 (TLR3) recognizes viral double-stranded RNA and its synthetic analog polyriboinosinic:polyribocytidylic acid (poly(I:C)) and induce inflammatory cytokines and dendritic cell activation (for review see [19]). Poly(I:C) has also been used in clinical trials and shown to have minimal toxicity[20]. Thus, poly(I:C) can potentially be used in combination with peptide-based vaccines to activate DCs and thus enhance the antigen-specific immune responses in humans.

In the current study, we explored the combination of an E7 peptide-based vaccine with PADRE peptide and poly(I:C) in the generation of E7-specific T cell immune responses and therapeutic antitumor effects. We observed that mice vaccinated with E7 peptide-based vaccine in combination with PADRE peptide and poly(I:C) generate significantly higher frequency of E7-specific CD8^+ ^T cells as well as significant therapeutic anti-tumor effects against TC-1 tumors. Furthermore, we found that intratumoral vaccination with the E7 peptide-based vaccine in combination with PADRE peptide and poly(I:C) generates even higher frequency of E7-specific CD8^+ ^T cells as well as better survival compared to subcutaneous vaccination in treated mice.

## Materials and methods

### Mice

Female C57BL/6 mice (5-8 weeks old) were purchased from the National Cancer Institute (Frederick, MD) and maintained under specific pathogen-free conditions in the oncology animal facility of the Johns Hopkins Hospital (Baltimore, MD). Animals were used in compliance with institutional animal health care regulations, and all procedures were performed according to the Johns Hopkins Institutional Care and Use Committee approved protocols.

### Cells and antibodies

TC-1 cells, which are an E7-expressing murine tumor model, were obtained by co-transformation of primary C57BL/6 mouse lung epithelial cells with HPV-16 E6 and E7 and an activated ras oncogene as previously described [21]. They were maintained in RPMI medium (Invitrogen, Carlsbad, CA, USA) supplemented with 2 nM glutamine, 1 mM sodium pyruvate, 20 mM HEPES, 50 μM β-mercaptoethanol, 100 IUml^-1 ^penicillin, 100 μg ml^-1 ^streptomycin and 10% fetal bovine serum (FBS) (Gemini Bio-Products, Woodland, CA, USA). Anti-mouse CD8a mAb (clone 53.6.7), CD4 (cloneGK1.5) and IFN-γ (BD Pharmingen, San Diego, CA, USA) were used for intracellular cytokine analysis.

### Peptide vaccination

Peptide vaccines were prepared by different mixtures of HPV-16 E7 (aa 49-57) peptide (RAHYNIVTE, 20 μg), PADRE peptide (AKFVAAWTLKAAA, 20 μg), E7(aa 49-57)-PADRE fusion peptide (RAHYNIVTEAKFVAAWTLKAAA, 20 ug) and polyriboinosinic:polyribocytidylic acid (poly IC, 20 μg) (Sigma-Aldrich^®^, USA) within 100 μl of PBS. C57BL/6 mice were immunized either subcutaneously at the inguinal area or directly into the TC-1 tumor mass every week for 2 continuous weeks or longer.

### Intracellular cytokine staining and flow cytometry analysis

Splenocytes were harvested from mice 1 week after their second vaccination. Prior to intracellular cytokine staining, 5×10^6 ^pooled splenocytes were incubated overnight with 1 μg ml^-1 ^E7 peptide (aa 49-57) or PADRE peptide (AKFVAAWTLKAAA) in the presence of GolgiPlug (BD Pharmingen, San Diego, CA) (1 μg ml^-1 ^). The stimulated splenocytes were then washed once with FACScan buffer and stained with phycoerythrin-conjugated monoclonal rat anti-mouse CD8a or CD4. Cells were subjected to intracellular cytokine staining using the Cytofix/Cytoperm kit according to the manufacturer's instructions (BD Pharmingen). Intracellular IFN-γ was stained with fluorescein isothiocyanate-conjugated rat anti-mouse IFN-γ to identify the immune response and cytokine levels. Flow cytometry analysis was performed using FACSCalibur with CELLQuest software (BD Biosciences, MountainView, CA, USA).

### In vivo tumor treatment experiments

5-8-week-old C57BL/6 mice (5 per group) were challenged subcutaneously with 1×10^4^/mouse of TC-1 tumor cells. In general, mice injected with 10^4 ^TC-1 tumor cells will develop tumor in 100% cases. In addition, if left untreated, the tumor will eventually kill the mice within 2 months. Three days after tumor challenge, the mice were immunized subcutaneously using 20 μg/mouse of HPV-16 E7 (aa 49-57) peptide, 20 μg/mouse of PADRE peptide or a mixture of E7 and PADRE peptide (20 μg each) or the E7(aa49-57)-PADRE fusion peptide (20 μg/mouse) with or without treatment with 20 μg/mouse of poly(I:C). The mice were given booster with the same dose every week at the same site until they died or the tumor reaches 2 cm in diameter. Tumor growth was monitored twice a week by inspection and palpation.

### Evaluation of tumor infiltrating lymphocytes

TC-1 tumors were harvested from euthanized mice after the skin was disinfected and carefully dissected. Medium (5 ml) was added and the tumor was disintegrated by tweezers by rubbing against the mesh thus releasing the entrapped lymphocytes. Cells were then filtered and treated with AKT lysing buffer (Quality Biological, INC. MD, USA) before intracellular staining.

### Statistical Analysis

All data expressed as means ± standard deviation (s.d.) are representative of at least two different experiments. Comparisons between individual data points for tumor sizes were made using a Student's *t*-test or repeated measure ANOVA (analysis of variance) test. Differences in survival between experimental groups were analyzed using the log rank test. Tumor sizes were calculated using the following equation: (tumor length × width × height)/2. Death of mouse was arbitrarily defined as tumor diameter greater than 2 cm.

## Results

### Mice vaccinated with the E7 peptide in combination with PADRE peptide and poly(I:C) generate the highest frequency of E7-specific CD8^+ ^T cells

**Figure 1:**
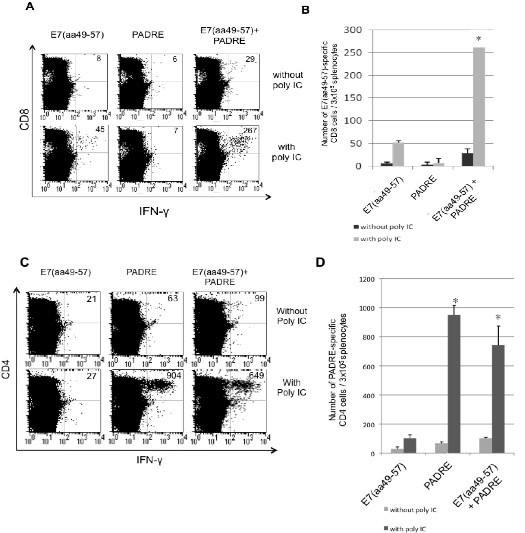
**Characterization of the number of E7-specific CD8+ T cells and PADRE-specific CD4+ T cells in vaccinated mice**. C57BL/6 mice (5 per group) were immunized subcutaneously using 20 μg/mouse of HPV-16 E7 (aa 49-57) peptide, 20 μg/mouse of PADRE peptide or a combination of the two with or without treatment with 20 μg/mouse of poly(I:C). Mice received a booster dose one week later. One week after the last vaccination, splenocytes from vaccinated mice were harvested and stimulated with the E7 or PADRE peptide. Cells were characterized for E7-specific CD8^+ ^T cells or PADRE-specific CD4+ T cells using intracellular IFN-*γ *staining followed by flow cytometry analysis. Splenocytes without peptide stimulation were used as negative control. **(A) **Representative data of intracellular cytokine staining followed by flow cytometry analysis showing the number of E7-specific IFNγ+ CD8+ T cells in the various groups (right upper quadrant). **(B) **Bar graph depicting the numbers of E7-specific IFN-γ-secreting CD8^+ ^T cells per 3 × 10^5 ^pooled splenocytes (mean ± s.d.). **(C) **Representative data of intracellular cytokine staining followed by flow cytometry analysis showing the number of PADRE-specific IFNγ+ CD4+ T cells in the various groups (right upper quadrant). **(D) **Bar graph depicting the numbers of PADRE-specific IFN-γ-secreting CD4^+ ^T cells per 3 × 10^5 ^pooled splenocytes (mean ± s.d.). Data shown are representative of two experiments performed. * indicates *p *< 0.05.

In order to determine the antigen-specific T cell immune responses in mice vaccinated with the combination of E7 peptide-based vaccine with PADRE peptide and poly(I:C), we performed intracellular cytokine staining followed by flow cytometry analyses. C57BL/6 mice (5 per group) were immunized subcutaneously with the E7 and/or PADRE peptide-based vaccine with or without poly(I:C) twice with a 1-week interval. One week after the last vaccination, splenocytes from vaccinated mice were harvested and characterized for E7-specific CD8^+ ^T cells (Figure 1A and 1B) or PADRE-specific CD4+ T cells (Figure 1C and 1D) using intracellular IFN-*γ *staining followed by flow cytometry analysis. As observed in Figure 1A and 1B, we found that mice vaccinated with the E7 peptide in combination with PADRE peptide and poly(I:C) generated a significantly higher number of E7-specific IFN-γ secreting CD8^+ ^T cells compared to mice vaccinated with E7 peptide with PADRE alone or poly(I:C) alone (* p < 0.05). In addition, we observed that mice vaccinated with PADRE peptide with or without E7 peptide in combination with poly(I:C) also generated significant increase in the number of PADRE-specific CD4+ T cells compared to mice vaccinated without poly(I:C) (Figure 1C and 1D). Furthermore, mice vaccinated with E7 peptide in combination with PADRE peptide and poly(I:C) generated the highest number of E7-specific IFN-γ secreting CD8^+ ^T cell immune response among all the vaccination groups. Thus, our data indicates that vaccination with the mixture of E7 and PADRE peptide in combination with poly(I:C) are capable of generating the best E7 peptide-specific T cell immune responses in vaccinated mice.

**Figure 2:**
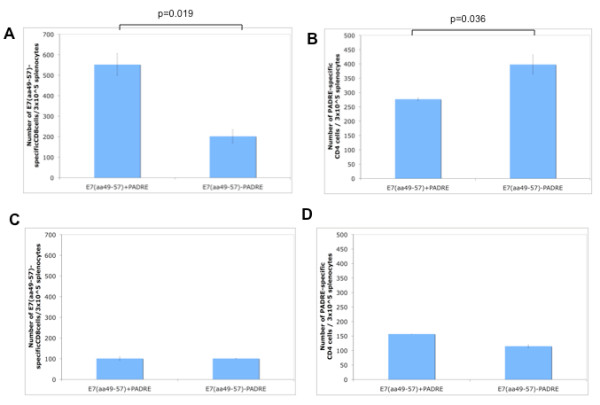
**Comparison of the number of E7-specific CD8+ T cells and PADRE-specific CD4+ T cells in mice vaccinated with the E7 and PADRE mixture with poly(I:C) versus the E7-PADRE fusion peptide with poly(I:C)**. C57BL/6 mice (5 per group) were immunized subcutaneously using the mixture of E7 and PADRE peptide with poly(I:C) or the E7+PADRE fusion peptide with poly(I:C). Mice received a booster dose one week later. One week **(**A & B**) **or 5 weeks **(C & D)** after the last vaccination, splenocytes from vaccinated mice were harvested and stimulated with the E7 or PADRE peptide. Cells were characterized for E7-specific CD8^+ ^T cells or PADRE-specific CD4+ T cells using intracellular IFN-*γ *staining followed by flow cytometry analysis. Splenocytes without peptide stimulation were used as negative control. **(A) **Bar graph depicting the numbers of E7-specific IFN-γ-secreting CD8^+ ^T cells per 3 × 10^5 ^pooled splenocytes (mean ± s.d.). **(B) **Bar graph depicting the numbers of PADRE-specific IFN-γ-secreting CD4^+ ^T cells per 3 × 10^5 ^pooled splenocytes (mean ± s.d.). **(C) **Bar graph depicting the numbers of memory E7-specific CD8^+ ^T cells per 3 × 10^5 ^pooled splenocytes (mean ± s.d.). **(D) **Bar graph depicting the numbers of memory PADRE-specific CD4^+ ^T cells per 3 × 10^5 ^pooled splenocytes (mean ± s.d.). Data shown are representative of two experiments performed.

We further compared the antigen-specific T cell immune responses generated by vaccination with the mixture of E7 and PADRE peptide with poly(I:C) and the E7-PADRE fusion peptide with poly(I:C). Our data indicate that the mixture of E7 and PADRE peptide generates significantly better E7-specific CD8+ T cell immune responses compared to the E7-PADRE fusion peptide (Figure 2A). In comparison, the E7 and PADRE mixture generates significantly lower PADRE-specific CD4+ T cell immune responses compared to the E7-PADRE fusion peptide (Figure 2B).

We also characterized the long-term memory immune responses generated by vaccination with the mixture of E7 and PADRE peptide and the E7-PADRE fusion peptide. We found that there was no significant difference in the E7-specific CD8+ T cell immune responses and the PADRE-specific CD4+ T cell immune responses generated by the mixture of E7 and PADRE peptide and the E7+PADRE fusion peptide (Figure 2C and 2D). Taken together, our data indicates that vaccination with the mixture of E7 peptide with PADRE peptide in combination with poly(I:C) leads to significantly higher peptide-specific immune responses compared to vaccination with the E7-peptide fusion peptide.

### Treatment with the E7 peptide in combination with PADRE peptide and with poly(I:C) leads to better survival in TC-1 tumor-bearing mice

**Figure 3:**
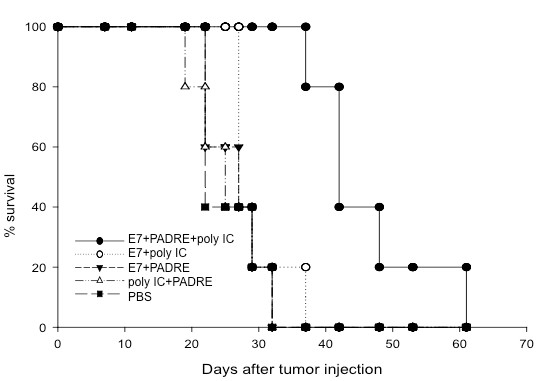
***In vivo *tumor treatment experiments**. C57BL/6 mice (5 per group) were challenged subcutaneously with 1×10^4^/mouse of TC-1 tumor cells. Three days later, the mice were immunized subcutaneously using HPV-16 E7 (aa 49-57) peptide with PADRE peptide or with poly(I:C) or with both poly(I:C) and PADRE peptide. Tumor-bearing mice treated with PBS or with poly(I:C) and PADRE without E7 peptide were used as controls. The mice were given booster with the same dose every week at the same site until they died or the tumor reaches 2 cm in diameter. The survival of tumor-bearing mice was analyzed by Kaplan & Meier analysis. Linear graph depicting survival of TC-1 tumor bearing mice treated with the combination of E7 and PADRE peptide with or without poly(I:C) (*p *< 0.05). Data shown are representative of two experiments performed.

In order to determine if tumor-bearing mice treated with E7 peptide in combination with PADRE peptide and poly(I:C) can demonstrate therapeutic antitumor effects, we performed in vivo tumor treatment experiments. C57BL/6 mice (5 per group) were challenged subcutaneously with TC-1 tumor cells in the right leg. Three days later, mice were immunized subcutaneously with the HPV-16 E7 (aa 49-57) peptide with PADRE peptide or with poly(I:C) or with both poly(I:C) and PADRE peptide at 1-week intervals. Tumor-bearing mice treated with PBS or with poly(I:C) and PADRE without E7 peptide were used as controls. Tumor growth was monitored twice a week by inspection and palpation. As shown in Figure 3, treatment with E7 peptide in combination with PADRE peptide and poly(I:C) demonstrated significantly better survival in TC-1 tumor-bearing mice compared to treatment with E7 peptide with PADRE alone or poly(I:C) alone (p < 0.05). Thus, our data indicate that treatment with the E7 peptide in combination with PADRE peptide and poly(I:C) leads to better survival in TC-1 tumor-bearing mice.

### Intratumoral vaccination with the E7 peptide in combination with PADRE peptide and poly(I:C) generates significantly higher frequency of E7-specific CD8^+ ^T cells compared to subcutaneous vaccination

**Figure 4:**
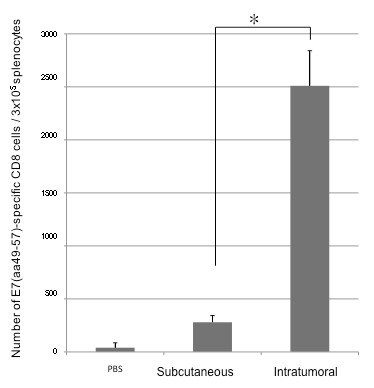
**Flow cytometry analysis to determine the number of E7-specific CD8+ T cells in the splenocytes of mice vaccinated subcutaneously or intratumorally**. The TC-1 tumor-bearing C57BL/6 mice (5 per group) were immunized subcutaneously or intratumorally using a combination of 20 μg/mouse of HPV-16 E7 (aa 49-57) peptide and 20 μg/mouse of PADRE peptide with 20 μg/mouse of poly(I:C) twice with a 1-week interval. One week after the last vaccination, splenocytes were harvested and characterized for E7-specific CD8^+ ^T cells using intracellular IFN-*γ *staining followed by flow cytometry analysis. **(A) **Representative flow cytometry data showing the number of E7-specific IFNγ+ CD8+ T cells in splenocytes from mice vaccinated subcutaneously or intratumorally (right upper quadrant). **(B) **Bar graph depicting the numbers of E7-specific IFN-γ-secreting CD8^+ ^T cells per 3 × 10^4 ^pooled splenocytes (mean ± s.d.). * indicates p < 0.05. Data shown are representative of two experiments performed.

In order to determine whether intratumoral vaccination with the E7 peptide in combination with PADRE peptide and poly(I:C) would generate enhanced E7-specific CD8+ T cell immune responses, C57BL/6 mice (5 per group) were challenged subcutaneously with TC-1 tumor cells in the right leg. Three days later, mice were immunized subcutaneously or intratumorally with the E7 and PADRE peptide-based vaccine with poly(I:C) twice with a 1-week interval. One week after the last vaccination, splenocytes from vaccinated mice were harvested characterized for E7-specific CD8^+ ^T cells using intracellular IFN-*γ *staining followed by flow cytometry analysis. As shown in Figure 4, mice vaccinated intratumorally with the E7 peptide in combination with PADRE peptide and poly(I:C) generated a significantly higher number of E7-specific IFN-γ secreting CD8^+ ^T cells in the splenocytes compared to mice vaccinated subcutaneously with the same vaccine regimen (* p < 0.05).

**Figure 5:**
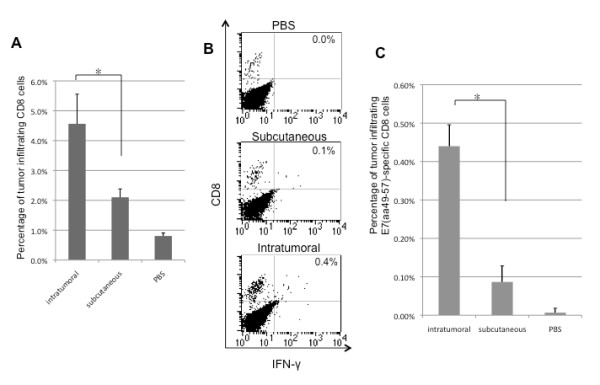
**Flow cytometry analysis to determine the number of E7-specific CD8+ T cells in the tumor-infiltrating lymphocytes of mice vaccinated subcutaneously or intratumorally**. The TC-1 tumor-bearing C57BL/6 mice (5 per group) were immunized subcutaneously or intratumorally using a combination of 20 μg/mouse of HPV-16 E7 (aa 49-57) peptide and 20 μg/mouse of PADRE peptide with 20 μg/mouse of poly(I:C) twice with a 1-week interval. One week after the last vaccination, TILs were harvested and characterized for E7-specific CD8^+ ^T cells using intracellular IFN-*γ *staining followed by flow cytometry analysis. **(A) **Bar graph depicting the numbers of tumor-infiltrating CD8^+ ^T cells from mice vaccinated either subcutaneously or intratumorally (mean ± s.d.). **(B) **Representative flow cytometry data showing the percentage of E7-specific IFNγ+ CD8+ T cells in tumor-infiltrating lymphocytes derived from mice vaccinated subcutaneously or intratumorally (right upper quadrant). **(C) **Bar graph depicting the numbers of tumor-infiltrating E7-specific CD8^+ ^T cells from mice vaccinated either subcutaneously or intratumorally (mean ± s.d.). Data shown are representative of two experiments performed. * indicates p < 0.05.

We then isolated the tumor-infiltrating lymphocytes from tumor-bearing mice vaccinated with E7 peptide in combination with PADRE peptide and poly(I:C) either intratumorally or subcutaneously and compared the immune responses. We observed that mice vaccinated intratumorally with E7 peptide in combination with PADRE peptide and poly(I:C) generated a significantly higher percentage of tumor-infiltrating CD8+ T cells (Figure 5A**) **as well as E7-specific CD8^+ ^T cells (Figure 5B and 5C) in the TILs compared to mice vaccinated with the same regimen subcutaneously (* p < 0.05). Taken together, our data indicates that intratumoral vaccination with E7 peptide in combination with PADRE peptide and poly(I:C) is capable of generating better E7-specific CD8+ T cell immune responses compared to subcutaneous vaccination.

### Tumor-bearing mice treated intratumorally with E7 peptide in combination with PADRE peptide and poly(I:C) demonstrate enhanced antitumor effects and prolonged survival

**Figure 6:**
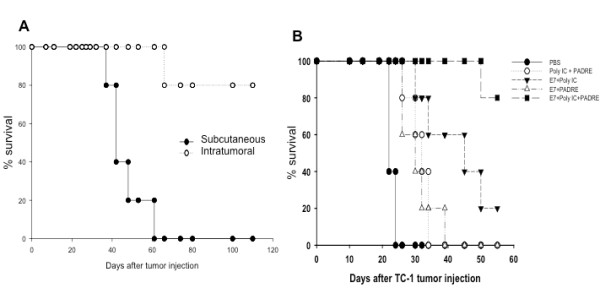
***In vivo *treatment experiments comparing subcutaneous vaccination and intratumoral vaccination**. **(A) **Kaplan-Meier graph depicting the survival of TC-1 tumor bearing mice treated either intratumorally or subcutaneously with the combination of E7 and PADRE peptide with poly(I:C). The TC-1 tumor-bearing C57BL/6 mice (5 per group) were immunized either subcutaneously or intratumorally using the combination of 20 μg/mouse of HPV-16 E7 (aa 49-57) peptide and 20 μg/mouse of PADRE peptide with 20 μg/mouse of poly(I:C). The mice were given one booster with the same peptide regimen and dose every week at the same site until they died or the tumor reaches 2 cm in diameter and survival was analyzed by Kaplan & Meier analysis. **(B) **Kaplan-Meier graph depicting survival of TC-1 tumor bearing mice treated intratumorally with the various combinations of reagents. Tumor-bearing C57BL/6 mice (5 per group) were treated via intratumoral injection using 20 μg/mouse of HPV-16 E7 (aa 49-57) peptide with 20 μg/mouse of PADRE peptide or with 20 μg/mouse of poly(I:C) or with both poly(I:C) and PADRE peptide. Tumor-bearing mice treated with PBS or with poly(I:C) and PADRE without E7 peptide were used as controls. The mice were given booster with the same dose every 5 days at the same site until they died or the tumor reaches 2 cm in diameter and survival was analyzed by Kaplan & Meier analysis. Data shown are representative of two experiments performed.

In order to determine if tumor-bearing mice treated intratumorally with E7 peptide in combination with PADRE peptide and poly(I:C) can demonstrate enhanced therapeutic antitumor effects compared to subcutaneous treatment, we performed in vivo tumor treatment experiments. C57BL/6 mice (5 per group) were challenged subcutaneously with TC-1 tumor cells in the right leg. Three days later, mice were immunized either subcutaneously or intratumorally with the E7 peptide-based vaccine in combination with PADRE peptide and poly(I:C) at 1-week intervals. As shown in Figure 6A, mice vaccinated intratumorally with the E7 peptide in combination with PADRE peptide and poly(I:C) demonstrated significantly better survival compared to mice vaccinated subcutaneously.

In order to compare the survival in tumor-bearing mice treated via intratumoral injection with the various reagents, tumor-bearing mice were treated via intratumoral injection using HPV-16 E7 (aa 49-57) peptide with PADRE peptide or with poly(I:C) or with both poly(I:C) and PADRE peptide. Tumor-bearing mice treated with PBS or with poly(I:C) and PADRE without E7 peptide were used as controls. The mice were given booster with the same dose every 5 days at the same site until they died or the tumor reaches 2 cm in diameter. The survival of tumor-bearing mice was analyzed by Kaplan & Meier analysis. As shown in Figure 6B, tumor-bearing mice vaccinated intratumorally with the E7 peptide in combination with PADRE peptide and poly(I:C) demonstrated significantly better survival compared to mice treated with E7 peptide with PADRE alone or E7 peptide with poly(I:C) alone (p < 0.05). Taken together, our data indicate that intratumoral vaccination with the E7 peptide in combination with PADRE peptide and poly(I:C) generates significantly enhanced therapeutic anti-tumor effects against TC-1 tumors.

## Discussion

In the current study, we observed that mice vaccinated with the E7 peptide-based vaccine combined with PADRE peptide and poly(I:C) generate the strongest E7-specific CD8^+ ^T cell immune responses and therapeutic anti-tumor effects against TC-1 tumors among the different vaccination groups. Furthermore, we found that intratumoral vaccination with the E7 peptide-based vaccine in combination with PADRE peptide and poly(I:C) generates significantly higher frequency of E7-specific CD8^+ ^T cells as well as better survival compared to subcutaneous vaccination with the same regimen in treated mice.

We observed that the inclusion of PADRE peptide could significantly improve the E7-specific immune responses generated by the E7 peptide-based vaccine in vaccinated mice. Our data is consistent with our previous studies using DNA-based vaccines. We have previously employed a DNA vaccine encoding an invariant (Ii) chain in which the CLIP region is replaced with the PADRE epitope (Ii-PADRE) [22]. We demonstrated that mice vaccinated with DNA encoding Ii-PADRE showed significantly greater PADRE-specific CD4^+ ^T cell immune responses compared to mice vaccinated with DNA encoding Ii chain alone [22]. More importantly, co-administration of DNA encoding HPV E7 antigen with Ii-PADRE DNA led to significantly higher frequency of E7-specific CD8^+ ^T cell immune responses and more potent protective and therapeutic antitumor effects against TC-1 tumors in treated mice [22]. Thus, the induction of CD4+ T help by employment of the PADRE strategy may be used in combination with DNA or peptide-based vaccination in order to enhance the antigen-specific immune responses and antitumor effects.

In our study, we found that intratumoral administration of the E7 peptide-based vaccines in conjunction with poly(I:C) generated significantly greater E7-specific immune responses and antitumor effects compared to subcutaneous vaccination (See Figures 4, 5 and 6). A potential mechanism for the observed effect may be related to the fact that the E7 CTL peptide may directly bind to MHC class I molecule of tumor cells, thus rendering them more susceptible to direct killing by E7-specific CD8+ T cells. This may also result in release of E7 antigen from the apoptotic tumor cells which may be taken up by antigen-presenting cells, resulting in further presentation of E7 antigen to CD8+ T cells (so called cross-priming mechanism). These mechanisms may potentially contribute to the observed enhancement in the E7-specific CD8+ T cell immune responses and antitumor effects against E7-expressing tumors.

Another important mechanism for the observed enhancement in immune responses and antitumor effects by intratumoral administration of the peptide-based vaccine with poly(I:C) may be related to the alteration of the tumor microenvironment. Poly(I:C) has previously been shown to trigger the maturation of DCs and promote the production of inflammatory Th1 cytokines such as IL-12, while suppressing Th2 cytokines, such as IL-10 in vitro [23,24]. Thus, we speculate that intratumoral administration of peptide-based vaccines with poly(I:C) may potentially generate an Th1 anti-tumor inflammatory response in the tumor microenvironment, thus contributing to the destruction of the tumor. Furthermore, the released tumor antigen, such as E7 may potentially be taken up by antigen-presenting cells, leading to further activation of tumor-specific CD8+ T cells (cross-priming mechanism). Thus, intratumoral administration of poly(I:C) with the peptide-based vaccine may alter the tumor microenvironment to enhance the E7-specific immune responses as well as antitumor effects generated by the E7 peptide-based vaccine.

In summary, our study demonstrates that intratumoral administration of an E7-peptide-based vaccine in combination with PADRE peptide and poly(I:C) leads to enhanced antitumor effects in treated mice. The employment of intratumoral administration of the peptide-based vaccines in conjunction with PADRE peptide and poly(I:C) can potentially be applied for advanced cervical tumors which are not surgically resectable to improve the clinical outcome. However, this approach is restricted to a particular E7 peptide. For future clinical translation, we would require the employment of long overlapping peptides to overcome the limitation of MHC restriction and include more E7 CTL epitopes. Recent studies employing peptide vaccination using an overlapping set of long peptides comprising the sequences of the HPV16 E6 and E7 oncoproteins have been shown to demonstrate significant tumor-specific immune responses [25-27]. Thus, the employment of PADRE peptide and poly(I:C) may potentially be used in combination with overlapping peptide-based vaccines to enhance the antigen-specific immune responses and antitumor effects for the control of HPV-associated malignancies.

## Competing interests

The authors declare that they have no competing interests.

## Authors' contributions

CYW was involved in the execution of the project. AM was involved in the interpretation of the data and writing the manuscript. XP participated in the design of the study and the statistical analysis. CFH and TCW provided overall supervision and guidance for the project. All authors read and approved the manuscript.
